# Pretreatment serum lactate dehydrogenase is an independent prognostic factor for patients receiving neoadjuvant chemotherapy for locally advanced cervical cancer

**DOI:** 10.1002/cam4.779

**Published:** 2016-06-28

**Authors:** Jing Li, Miao‐Fang Wu, Huai‐Wu Lu, Qing Chen, Zhong‐Qiu Lin, Li‐Juan Wang

**Affiliations:** ^1^ Department of Gynecologic OncologySun Yat‐Sen Memorial HospitalSun Yat‐Sen UniversityGuangzhou510120China; ^2^ Team‐Based Learning Group of Clinical StudySun Yat‐Sen UniversityGuangzhou510000China

**Keywords:** Cervical cancer, lactate dehydrogenase, locally advanced cancer, prognosis

## Abstract

For locally advanced cervical cancer (LACC), hypoxia is a characteristic property. This study aimed to investigate whether baseline lactic dehydrogenase (LDH) level, which is a marker of hypoxia, had clinical value in determining neoadjuvant chemotherapy (NACT) response and prognosis for LACC patients. The study cohort included 418 patients with a median follow‐up of 37.5 months. Cox proportional hazards models were used to assess the prognostic value of baseline LDH levels. Multivariate logistic regression analysis was performed to identify independent predictors of complete response after NACT. Backward stepwise selection with the Akaike information criterion was used to identify factors that could be entered into the multivariate regression model. Compared with patients with LDH levels <252.0 *μ*/L, patients with LDH levels ≥252.0 *μ*/L were more likely to have an elevated level of squamous cell carcinoma antigen, lymphatic vascular space involvement, lymph node metastasis, and positive parametrium and achieved lower complete remission rates. Baseline LDH levels ≥252.0 *μ*/L was an independent prognosticator for recurrence‐free survival (adjusted hazard ratio [HR], 3.56; 95% confidence interval [CI] 2.22–5.69; *P *<* *0.0001) and cancer‐specific survival (adjusted HR, 3.08; 95% CI, 1.89–5.01; *P *<* *0.0001). The predictive value of baseline LDH value remained significant in the subgroup analysis. LDH level ≥252.0 *μ*/L was identified as an independent predictor of complete remission after NACT (adjusted odds ratio [OR], 0.29; 95% CI, 0.15–0.58; *P *<* *0.0001). Baseline LDH ≥252.0 *μ*/L is an independent prognostic predictor for patients receiving neoadjuvant chemotherapy for LACC. It helps distinguish patients with different prognosis and select patients who are more likely to benefit from NACT.

## Background

Cervical cancer is the leading cause of cancer‐related deaths in women in developing countries 1. In China, there were an estimated 87,982 new cases of cervical cancer and 23,375 related deaths in 2011 2. The treatment of cervical cancer is primary based on the stage of disease. Although concurrent chemoradiotherapy (CCRT) has been the standard of care since 1999, optimal management of bulky IB2 and IIA2 disease remains controversial 3. In this clinical setting, neoadjuvant chemotherapy (NACT) combined with radical hysterectomy is considered as an alternative therapeutic option. NACT can reduce tumor size thereby transforming inoperable tumors into radically resectable ones 4. Moreover, by decreasing the risk of lymph node metastasis, NACT can minimize the need for postsurgical radiotherapy. Additionally, a published meta‐analysis shows that NACT followed by surgery is superior to radiotherapy alone in terms of overall survival 5. Because of these advantages, NACT is used in up to 25% of cervical cancer patients in many parts of the world, such as Asia, Italy, and South America 6. On the other hand, there are several objections because for patients with locally advanced cervical cancer (LACC) who do not response to NACT, the delay in curative treatment, the development of radio‐resistant cellular clones and cross‐resistance with radiotherapy may exert negative impact on patient survival 7. Given these controversies, NACT is not recommended as a routine treatment for LACC patients in current National Comprehensive Cancer Network (NCCN) Clinical Practice guideline 1. The discrepancy raises the possibility that NACT may improve antitumor outcomes in a subset of patients. Therefore, appropriate biomarkers are needed to select patients who are most likely to benefit from such treatment.

For locally advanced solid tumors, hypoxia is a characteristic property due to rapid cancer cell proliferation, high metabolic demands, and functional angiogenesis 8. There is clear evidence that hypoxia can promote cancer development and it is involved in the resistance to treatment via the formation of new blood vessels 9. Lactate dehydrogenase (LDH) is known to be a marker of hypoxia, which plays an important role in the proliferation and metastasis of tumor cells 10. Moreover, pretreatment serum LDH levels have been found to correlate with the prognosis of patients with malignant diseases 11. However, to the best of our knowledge, the clinical significance of LDH has been never investigated in patients with LACC. Therefore, we conducted a large cohort study to investigate the prognostic and predictive value of LDH levels for patients treated with NACT for LACC.

## Materials and Methods

### Patients

After Institutional Review Board (IRB) approval was obtained at the Sun Yat‐Sen Memorial Hospital, the institutional database was utilized to identify cases. The medical records of all women who received NACT and subsequent class III radical hysterectomy for cervical cancer between January 2005 and June 2010 were reviewed. Patients were reclassified based on the FIGO (Federation International of Gynecology and Obstetrics) 2014 staging system 4. Inclusion criteria were as follows: histologically confirmed squamous cell carcinoma and adenocarcinoma, FIGO stage IB2 and IIA2 disease, blood collection for LDH measurements prior to NACT, and signed informed consent provided. Exclusion criteria were as follows: patients not completing the planned cycles of NACT, patients not receiving radical surgery after NACT, and patients receiving any previous treatment for cervical or uterine malignancies. Data collected included demographic information; operative, chemotherapy, and radiotherapy notes; histopathologic reports; and follow‐up notes.

Pretreatment evaluation included physical and gynecologic examination, chest radiography, pelvic ultrasonography, and laboratory tests. Further investigation was performed when indicated. Gynecologic examination was carried out by at least two senior gynecologists. Maximum tumor diameter was determined by clinical measurement. Two authorized pathologists from our institution who were blinded to study outcomes reviewed all cervical pathology.

The NACT regimens were employed as follows: TP, paclitaxel+cisplatin; FP, 5‐fluouracil+cisplatin; TC, paclitaxel+carboplatin; and BVP, bleomycin+vincristine+cisplatin. All patients underwent type III radical hysterectomy according to the Piver–Rutledge classification with pelvic lymphadenectomy within 4 weeks after the last cycle of NACT. Pathological responses were retrospectively evaluated, and a complete response (CR) was defined as no evidence of viable tumor cells on the tumorous area 12. CCRT was performed if patients had the following risk factors: positive parametrium, positive lymph nodes, involved surgical margins, greater than one‐third stromal invasion, and lymphatic vascular space involvement (LVSI) 4.

Blood samples were collected for laboratory tests within 1 week before initiation of NACT. Serological LDH levels were measured using a Hitachi Automatic Analyzer 7600‐020 (Hitachi High‐Technologies, Tokyo, Japan). Normal serum LDH enzyme activities were defined to 108.0–252.0 *μ*/L. Based on pretreatment serum LDH levels, patients were classified into high LDH group (HL group, LDH ≥252.0 *μ*/L) and normal LDH group (NL group, LDH <252.0 *μ*/L). Serum squamous cell carcinoma antigen (SCCA) was assessed with an immunoradiometric assay kit (Imx; Abbott Diagnostics, Abbott Park, IL). A cut‐off value of 3.5 ng/mL was used to stratify patients into normal and abnormal group 13. The intraassay variation was <5% for all variables measured. Laboratory personnel performing these assays were blinded to study outcomes.

Follow‐up visit including complete history and physical examination and Papanicolau smear of the vaginal vault was recommended every 3 months for 2 years, every 6 months for the next 3 years, and once per year thereafter. Follow‐up information was obtained from office visits or telephone interviews. Tumor recurrence was diagnosed on the basis of clinical symptoms, physical examinations, biopsy, and imaging methods including positron emission tomography‐computed tomography (PET‐CT), magnetic resonance imaging (MRI), and computed tomography (CT). The primary endpoint of this study was to investigate whether the baseline serum LDH was a prognostic factor for recurrence‐free survival (RFS) and cervical cancer‐specific survival (CSS). RFS was measured from the date of NACT until the date of recurrence or last follow‐up. CSS was calculated as the time interval between the date of NACT and the date of death from cervical cancer or the date of last follow‐up.

### Statistical analyses

Data were analyzed using STATA/SE version 12.0 statistical software (Stata Corp. LP, College Station, TX) and SPSS version 14.0 (SPPS Inc., Chicago, IL). Continuous variables were presented as the median and range, while Categorical variables were presented as the number and percentages. The Kolmogorov–Smirnov test was used to determine the distribution of continuous variables. Student's *t‐*test was used to compare normally distributed continuous variables, whereas Mann–Whitney *U* test was used for data with nonnormal distribution. Chi‐square test (*χ* ^*2*^) or Fisher exact test were used to analyze the frequency distribution between categorical variables where appropriate. RFS and CSS were estimated using the Kaplan–Meier method and compared with the log‐rank test. Multivariate analysis (enter method) was performed to identify independent predictors for survival outcomes with Cox proportional hazards model, and hazard ratios (HRs) and 95% confidence intervals (CIs) were presented. The assumption of proportional hazards was tested based on Schoenfeld residuals 14. A binary logistic regression model for multivariate analysis was also used to determine independent predictor for CR after NACT, expressed with odds ratio (OR), and 95% CI. Akaike information criteria with backward selection were used to select variables that were entered into the multivariate model. All statistical tests were two‐tailed, and a *P* value of <0.05 was considered to be statistically significant.

## Results

### Characteristics of the study population

The final study cohort included 418 patients with a median follow‐up of 37.5 months (range: 4–65 months). The median age at the diagnosis of cervical cancer was 52.0 years (range: 24–80 years). The median serum LDH level for the entire cohort was 194.0 *μ*/L with a range 63 to 634 *μ*/L. An overview of clinic‐pathologic characteristics of all patients is given in Table 1. Of the included patients, 322 (77.03%) had serum LDH levels <252.0 *μ*/L, whereas 96 (22.97%) had serum LDH levels ≥252.0 *μ*/L. Compared with patients in the NL group, patients in the HL group were more likely to have lymphatic vascular space involvement (LVSI) (65.6% vs. 52.2%, *P *=* *0.020, *r *=* *0.114), lymph node metastasis (59.4% vs. 29.8%, *P *<* *0.0001, *r *=* *0.258), and positive parametrium (9.4% vs. 2.2%, *P *<* *0.003, *r *=* *0.158). The proportion of patients who achieved CR after NACT was significantly lower in the HL group in comparison with those in the NL group (11.5% vs. 32.3%, *P *<* *0.0001, *r *=* *−0.196). More patients in the HL group complicated with anemia, although it did not reach statistical significance (60.4% vs. 49.1%, *P *=* *0.051, *r *=* *0.096).

**Table 1:** Baseline demographic characteristics

	LDH <252 *μ*/L (*n* = 322)	LDH ≥252 *μ*/L (*n* = 96)	*P* value
Age (years), median (range)	51 (24–80)	50 (27–71)	0.345
BMI (kg/m^2^), *n* (%)			
<25	276 (85.7)	85 (88.5)	0.479
≥25	46 (14.3)	11 (11.5)	
Smoking, *n* (%)			
Never	298 (92.5)	94 (97.9)	0.194
Former	11 (3.4)	0 (0)	
Current	4 (1.2)	1 (1.0)	
Missing data	9 (2.8)	1 (1.0)	
Regular screening, *n* (%)			
Yes	24 (7.5)	2 (2.1)	0.150
No	279 (86.6)	87 (90.6)	
Missing data	19 (5.9)	7 (7.3)	
SCCA, *n* (%)			
≥3.5 ng/mL	173 (53.7)	64 (66.7)	0.025
<3.5 ng/mL	149 (46.3)	32 (33.3)	
Stage, *n* (%)			
IB2	167 (51.9)	53 (55.2)	0.565
IIA2	155 (48.1)	43 (44.8)	
Tumor histology, *n* (%)			
SCC	272 (84.5)	77 (80.2)	0.323
NSCC	50 (15.5)	19 (19.8)	
Differentiation, *n* (%)			
1	174 (54.0)	50 (52.1)	0.612
2	110 (34.2)	31 (32.3)	
3	38 (11.8)	15 (15.6)	
Deep stromal invasion, *n* (%)			
Yes	253 (78.6)	79 (82.3)	0.429
No	69 (21.4)	17 (17.7)	
LVSI, *n* (%)			
Yes	168 (52.2)	63 (65.6)	0.020
No	154 (47.8)	33 (34.4)	
Positive margins, *n* (%)			
Yes	11 (3.4)	8 (8.3)	0.080
No	311 (96.6)	88 (91.7)	
Positive nodes, *n* (%)			
Yes	96 (29.8)	57 (59.4)	<0.0001
No	226 (70.2)	39 (40.6)	
Positive parametrium, *n* (%)			
Yes	7 (2.2)	9 (9.4)	0.003
No	315 (97.8)	87 (90.6)	
CCRT, *n* (%)			
Yes	264 (82.0)	92 (95.8)	0.001
No	58 (18.0)	4 (4.2)	
CR achieved, *n* (%)			
Yes	104 (32.3)	11 (11.5)	<0.0001
No	218 (67.7)	85 (88.5)	
HGB (g/L), *n* (%)			
≥110	164 (50.9)	38 (39.6)	0.051
<110	158 (49.1)	58 (60.4)	
NACT regimen, *n* (%)			
Cisplatin+paclitaxel	282 (87.6)	83 (86.5)	0.772
Cisplatin‐based	40 (12.4)	13 (13.4)	

BMI, body mass index; CCRT, concurrent chemoradiation; CR, complete response; HGB, hemoglobin; LDH, lactate dehydrogenase; LVSI, lymphatic vascular space involvement; NACT, neoadjuvant chemotherapy; NSCC, nonsquamous cell carcinoma; SCC, squamous cell carcinoma; SCCA, squamous cell carcinoma antigen.

### Survival analysis

**Figure 1:**
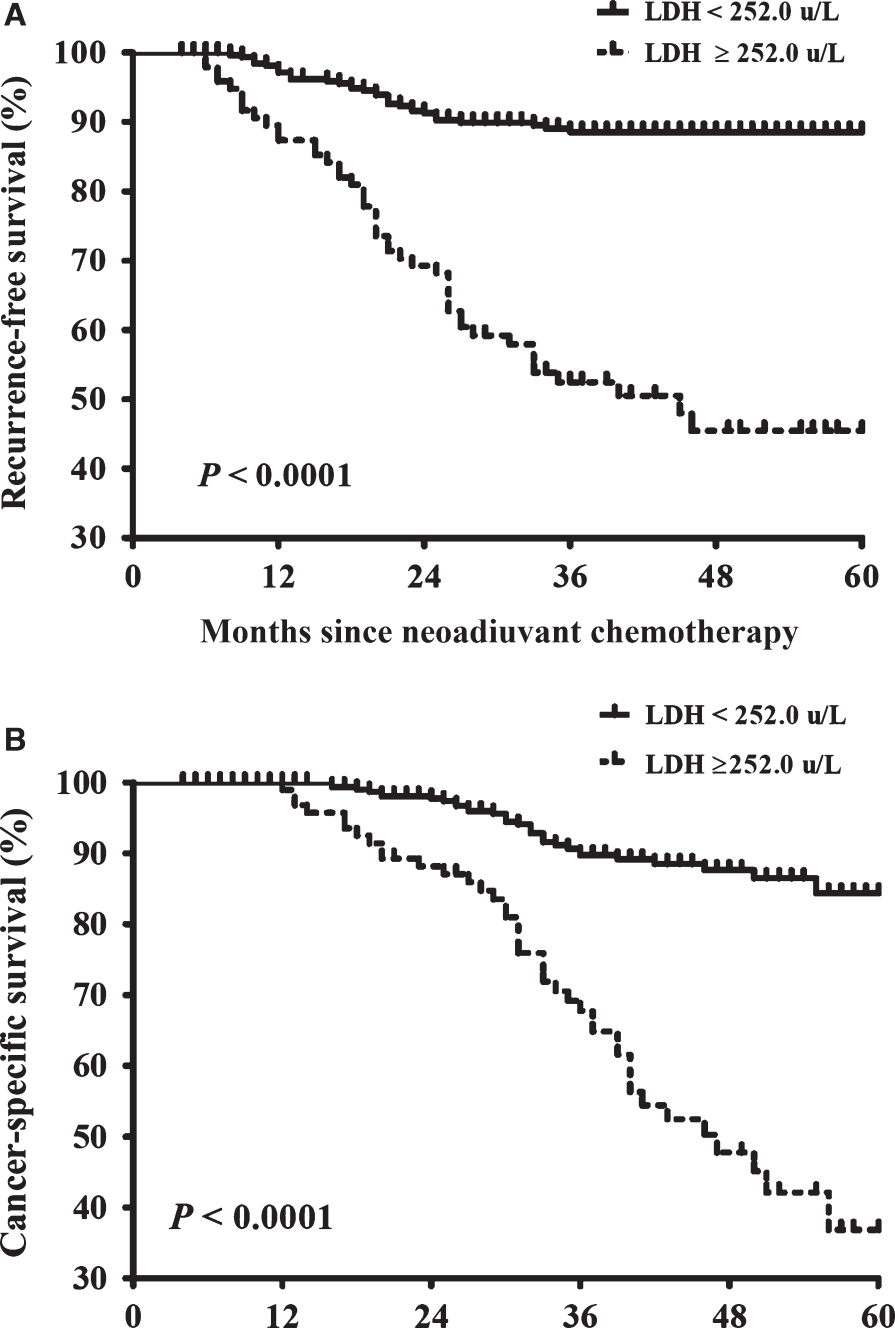
Kaplan–Meier survival curves for survival of cervical cancer patients receiving neoadjuvant chemotherapy for locally advanced disease. (A) recurrence‐free survival and (B) cancer‐specific survival. Patients were stratified by baseline lactic dehydrogenase levels. The *P* values were determined by the log‐rank test.

Overall, cancer recurrence was detected in 80 (19.2%) patients, consisting of 46 (47.9%) patients in the HL group and 34 (10.6%) patients in the NL group. In the subset of patients experiencing recurrence, the median interval between NACT and recurrence was 20 months (range: 6–46 months) and 90% of recurrence were identified within 3 years after NACT. The estimated 1‐, 3‐, and 5‐year recurrence rates for patients in the HL group were 12.5%, 44.8%, and 47.9%, compared with 2.8%, 10.6%, and 10.6% for those in the NL group, respectively. The proportional hazards assumption in the Cox model was assessed; overall, there was no evidence that the assumption was violated. The Kaplan–Meier curves and log‐rank test for RFS is illustrated in Figure 1A, which indicated that patients with an LDH level ≥252.0 *μ*/L had decreased RFS (*P *<* *0.0001). There were 73 (17.5%) patients died from cervical cancer, including 41 (42.7%) patients in the HL group and 32 (9.9%) patients in the NL group. The estimated 1‐, 3‐, and 5‐year cancer‐specific death rates for patients in the HL group were 9.4%, 21.9%, and 42.7%, compared with 0%, 8.4%, and 9.9% for those in the NL group, respectively. The Kaplan–Meier curves and log‐rank test for CSS are displayed in Figure 1B. A comparison of Kaplan–Meier curves for CSS showed patients with LDH levels ≥252.0 *μ*/L had significantly poor CSS (*P *<* *0.0001).

Tables 2 and 3 summarize the univariate and multivariate HR and 95% CI for RFS and CSS. On univariate analysis, pretreatment LDH level ≥252.0 *μ*/L was significantly associated with decreased RFS (HR, 5.47; 95% CI, 3.51–8.53; *P *<* *0.0001); other clinico‐pathological variables that significantly associated with decreased RFS included nonsquamous histology, deep stromal invasion, LVSI, positive surgical margins, node metastasis, and positive parametrium (all *P *<* *0.05). Achieving CR after NACT (HR, 0.16; 95% CI, 0.06–0.44; *P *<* *0.0001) and hemoglobin levels prior to NACT ≥110 g/L (HR, 0.93; 95% CI, 0.34–0.85; *P *=* *0.008) were associated with an improved RFS. By use of the backward selection with the AIC, we noted that nine variables were associated with RFS and LDH level ≥252.0 *μ*/L remained an independent prognostic factors for poor RFS (adjusted HR, 3.56; 95% CI, 2.22–5.69; *P *<* *0.0001). Other factors that were independently associated with decreased RFS included nonsquamous cell carcinoma (adjusted HR, 1.95; 95% CI, 1.18–3.20; *P *=* *0.009), positive surgical margins ((adjusted HR, 4.00; 95% CI, 2.11–7.61; *P *<* *0.0001), node metastasis (adjusted HR, 3.83; 95% CI, 2.20–6.65; *P *<* *0.0001), positive parametrium (adjusted HR, 2.73; 95% CI, 1.45–5.13; *P *=* *0.002), and SCCA levels ≥3.5 ng/mL (adjusted HR, 1.86; 95% CI, 1.17–2.96; *P = *0.008). On the other hand, CR after NACT (adjusted HR, 0.32; 95% CI, 0.13–0.82; *P = *0.018) was an independent predictors for an improved RFS.

**Table 2:** Univariate and multivariate cox proportional hazards regression analysis for recurrence‐free survival

	Univariate analysis			Multivariate analysis		
	HR	95% CI	*P* value	HR	95% CI	*P* value
Age (≥60 vs. <60)	0.97	0.52, 1.78	0.908			
BMI (≥25 kg/m^2^ vs. <25 kg/m^2^)	1.08	0.95, 1.22	0.246			
Histology (NSCC vs. SCC)	2.28	1.40, 3.70	0.001	1.95	1.18, 3.20	0.009
Tumor stage (IIA2 vs. IB2)	1.08	0.86, 1.34	0.517			
Tumor differentiation (G3 vs. G1–2)	1.00	0.51, 1.93	0.989			
Deep stromal invasion (yes vs. no)	1.97	1.02, 3.82	0.045			
LVSI (yes vs. no)	2.24	1.37, 3.66	0.001	1.47	0.88, 2.47	0.141
Positive margins (yes vs. no)	6.41	3.46, 11.88	<0.0001	4.00	2.11, 7.61	<0.0001
Positive nodes (yes vs. no)	6.53	3.90, 10.94	<0.0001	3.83	2.20, 6.65	<0.0001
Positive parametrium (yes vs. no)	8.45	4.63, 15.41	<0.0001	2.73	1.45, 5.13	0.002
CR achieved (yes vs. no)	0.16	0.06, 0.44	<0.0001	0.32	0.13, 0.82	0.018
LDH (≥252.0 *μ*/L vs. <252.0 *μ*/L)	5.47	3.51, 8.53	<0.0001	3.56	2.22, 5.69	<0.0001
SCCA (≥3.5 ng/mL vs. <3.5 ng/mL)	0.96	0.62, 1.50	0.869	1.86	1.17, 2.96	0.008
HGB (≥110 g/L vs. <110 g/L)	0.93	0.34, 0.85	0.008	0.72	0.46, 1.12	0.141

BMI, body mass index; CI, confidence interval; CR, complete response; HGB, hemoglobin; HR, hazard ratio; LDH, lactate dehydrogenase; LVSI, lymphatic vascular space involvement; NSCC, nonsquamous cell carcinoma; SCC, squamous cell carcinoma; SCCA, squamous cell carcinoma antigen.

**Table 3:** Univariate and multivariate cox proportional hazards regression analysis for cancer‐specific survival

	Univariate analysis			Multivariate analysis		
	HR	95% CI	*P* value	HR	95% CI	*P* value
Age (≥60 vs. <60)	0.71	0.34, 1.48	0.360			
BMI (≥25 kg/m^2^ vs. <25 kg/m^2^)	1.06	0.93, 1.21	0.358			
Histology (NSCC vs. SCC)	2.40	1.46, 3.96	0.001	1.90	1.13, 3.19	0.015
Tumor stage (IIA2 vs. IB2)	1.27	1.01, 1.60	0.044			
Tumor differentiation (G3 vs. G1–2)	1.17	0.60, 2.28	0.647			
Deep stromal invasion (yes vs. no)	2.10	1.05, 4.23	0.037			
LVSI (yes vs. no)	1.98	1.20, 3.27	0.007			
Positive margins (yes vs. no)	8.11	4.41, 14.93	<0.0001	4.40	2.32, 8.34	<0.0001
Positive nodes (yes vs. no)	6.28	3.68, 10.71	<0.0001	3.98	2.26, 7.01	<0.0001
Positive parametrium (yes vs. no)	10.07	5.43, 18.68	<0.0001	3.72	1.96, 7.04	<0.0001
CR achieved (yes vs. no)	0.14	0.05, 0.38	<0.0001	0.28	0.10, 0.80	0.018
LDH (≥252.0 *μ*/L vs. <252.0 *μ*/L)	4.92	3.10, 7.81	<0.0001	3.08	1.89, 5.01	<0.0001
HGB (≥110 g/L vs. <110 g/L)	0.92	0.58, 1.45	0.717	0.70	0.44, 1.12	0.135
SCCA (≥3.5 ng/mL vs. <3.5 ng/mL)	0.98	0.62, 1.56	0.937	1.76	1.10, 2.83	0.019

BMI, body mass index; CI, confidence interval; CR, complete response; HGB, hemoglobin; HR, hazard ratio; LDH, lactate dehydrogenase; LVSI, lymphatic vascular space involvement; NSCC, nonsquamous cell carcinoma; SCC, squamous cell carcinoma; SCCA, squamous cell carcinoma antigen.

On univariate analysis, an elevated level LDH ≥252.0 *μ*/L was significantly associated with poor CSS (adjusted HR, 4.92; 95% CI, 3.10–7.81; *P *<* *0.0001). In addition, as determined by univariate analysis, nonsquamous histology, FIGO stage IIA2 disease, deep stromal invasion, LVSI, positive surgical margins, node metastasis, and positive parametrium were significantly associated with a decreased CSS (all *P *<* *0.05), whereas CR after NACT was a favorable factor for an improved CSS (HR, 0.14; 95% CI, 0.05–0.38; *P *<* *0.0001). Based on AIC, eight variables were included in the multivariate Cox proportional hazards regression analysis. An LDH level ≥252.0 *μ*/L was an independent predictive risk factor for poor CSS (adjusted HR, 3.08; 95% CI, 1.89–5.01; *P *<* *0.0001). Additionally, nonsquamous histology (adjusted HR, 1.90; 95% CI, 1.13–3.19; *P *=* *0.015), positive surgical margins (adjusted HR, 4.40; 95% CI, 2.32–8.34; *P *<* *0.0001), node metastasis (adjusted HR, 3.98; 95% CI, 2.26–7.01; *P *<* *0.0001), positive parametrium (adjusted HR, 3.72; 95% CI, 1.96–7.04; *P *<* *0.0001), and SCCA levels ≥3.5 ng/mL (adjusted HR, 1.76; 95% CI, 1.10–2.83; *P *=* *0.019) were independently associated with poor CSS, whereas CR after NACT was an independent predictor for an improved CSS (adjusted HR, 0.28; 95% CI, 0.10–0.80; *P *=* *0.018).

### Subgroup analysis

We further evaluated the prognostic effects of LDH on RFS and CSS according to patient baseline characteristics, using a Cox proportional hazards regression model. Table 4 summarizes the results of subgroup analysis. The prognostic value of elevated LDH levels remained all subgroups.

**Table 4:** Subgroup analysis of adjusted hazard ratios of survival for LDH using the cox proportional hazard model

	No. of patients	Recurrence‐free survival			Cancer‐specific survival		
		HR	95% CI	*P* value	HR	95% CI	*P* value
Age							
<60	352	5.11	3.16, 8.27	<0.0001	4.04	2.48, 6.58	<0.0001
≥60	66	8.34	2.59, 26.85	<0.0001	22.76	4.43, 119.45	<0.0001
BMI (kg/m^2^)							
<25	361	5.69	3.53, 9.17	<0.0001	4.91	3.01, 8.01	<0.0001
≥25	57	4.34	1.26, 14.99	0.020	5.05	1.20, 21.27	0.027
Histology							
SCC	349	4.88	2.89, 8.22	<0.0001	4.46	2.57, 7.73	<0.0001
NSCC	69	6.93	2.92, 16.47	<0.0001	5.43	2.27, 12.97	<0.0001
Tumor stage							
IB2	220	4.39	2.35, 8.18	<0.0001	4.17	2.06, 8.47	<0.0001
IIA2	198	7.00	3.71, 13.22	<0.0001	5.83	3.16, 10.75	<0.0001
Tumor differentiation							
G1–2	365	6.29	3.90, 10.13	<0.0001	5.74	3.48, 9.47	<0.0001
G3	53	2.20	0.64, 7.62	0.212	1.80	0.51, 6.35	0.364
CR achieved							
No	303	4.75	2.99, 7.55	<0.0001	4.06	2.52, 6.56	<0.0001
Yes	115	2.31	0.36, 20.65	0.455	3.15	0.32, 30.72	0.324
HGB (g/L)							
<110	216	7.74	3.93, 15.24	<0.0001	5.52	2.96, 11.45	<0.0001
≥110	202	4.14	2.22, 7.73	<0.0001	4.40	2.28, 8.48	<0.0001
SCCA (ng/mL)							
<3.5	181	4.17	2.13, 8.16	<0.0001	3.46	1.68, 7.14	0.001
≥3.5	237	7.33	3.90, 13.80	<0.0001	6.86	3.57, 13.20	<0.0001
NACT regimen							
Cisplatin+paclitaxel	365	4.64	2.87, 7.51	<0.0001	4.53	2.69, 7.64	<0.0001
Cisplatin‐based	53	14.32	3.90, 52.69	<0.0001	8.53	2.83, 25.71	<0.0001

BMI, body mass index; CI, confidence interval; CR, complete response; HGB, hemoglobin; HR, hazard ratio; LDH, lactate dehydrogenase; NACT, neoadjuvant chemotherapy; NSCC, nonsquamous cell carcinoma; SCC, squamous cell carcinoma; SCCA, squamous cell carcinoma antigen.

### Factors associated with CR after NACT

CR after NACT has been confirmed as a reliable surrogate endpoint of survival for patients with LACC. Therefore, an additional logistic regression analysis was conducted to determine factors predicting CR after NACT, and the results are summarized in Table 5. At univariate analysis, pretreatment LDH levels ≥252.0 *μ*/L (OR, 0.27; 95% CI, 0.14–0.53; *P *<* *0.0001) and pretreatment SCCA levels ≥3.5 ng/mL (OR, 0.35; 95% CI, 0.23–0.55; *P *<* *0.0001) were significantly associated with decreased likelihood of CR after NACT. Four variables were included in the multivariate analysis according to stepwise selection based on AIC. Body mass index (BMI) ≥25 kg/m^2^ (OR, 0.35; 95% CI, 0.23–0.55; *P *<* *0.0001), LDH levels ≥252.0 *μ*/L (OR, 0.35; 95% CI, 0.23–0.55; *P *<* *0.0001), and SCCA levels ≥3.5 ng/mL (OR, 0.35; 95% CI, 0.23–0.55; *P *<* *0.0001) were independently associated with decreased incidence of CR after NACT.

**Table 5:** Univariate and multivariate analysis of variables associated with complete response after neoadjuvant chemotherapy

	Univariate analysis			Multivariate analysis^a^		
	OR	95% CI	*P* value	OR	95% CI	*P* value
Age (≥60 vs. <60)	0.99	0.55, 1.78	0.962			
BMI (≥25 kg/m^2^ vs. <25 kg/m^2^)	0.9	0.79, 1.03	0.114	0.41	0.19, 0.89	0.024
Histology (NSCC vs. SCC)	0.56	0.30, 1.07	0.081	0.54	0.27, 1.06	0.072
Tumor stage (IIA2 vs. IB2)	1.04	0.84, 1.29	0.738			
Tumor differentiation (G3 vs. G1–2)	0.94	0.49, 1.80	0.848			
LDH (>252.0 *μ*/L vs. <252.0 *μ*/L)	0.27	0.14, 0.53	<0.0001	0.29	0.15, 0.58	<0.0001
SCCA (≥3.5 ng/mL vs. <3.5 ng/mL)	0.35	0.23, 0.55	<0.0001	0.36	0.23, 0.57	<0.0001
NACT regimen (cisplatin+paclitaxel vs. cisplatin‐based)	1.05	0.55, 1.99	0.890			
HGB (≥110 g/L vs. <110 g/L)	0.73	0.48, 1.13	0.160			

BMI, body mass index; CI, confidence interval; HGB, hemoglobin; LDH, lactate dehydrogenase; NACT, neoadjuvant chemotherapy; NSCC, nonsquamous cell carcinoma; OR, odds ratio; SCC, squamous cell carcinoma; SCCA, squamous cell carcinoma antigen.

a Omnibus tests of model coefficients (enter method): *χ* ^2^ = 46.52, df* *= 4, *P *<* *0.0001; Hosmer–Lemeshow goodness‐of‐fit test for the fitted mode: *χ* ^2^ = 1.549, df = 5, *P *=* *0.907.

## Discussion

An inverse relationship between LDH levels and length of survival has also been identified in many tumor types, including melanoma 15, breast cancer 16, myeloma 17, hepatocellular carcinoma 18, seminoma 19, nasopharyngeal cancer 20, lung cancer 21, colorectal cancer 10, renal cancer 22, oral cancer 23, and pancreatic cancer 24. For gynecologic malignancies, a strong association between the elevated expression of LDH and an aggressive phenotype has been noted in patients with ovarian and uterine carcinoma 25, 26. In consistent with these findings, our study demonstrated that LACC patients with elevated LDH levels were more likely to have positive SCCA, LVSI, lymph node metastasis, and parametrium invasion. Furthermore, compared with patients with baseline LDH less than 252.0 *μ*/L, patients with LDH ≥252.0 *μ*/L had a statistically significant 3.6‐fold risk of cancer recurrence and 3.1‐fold risk of cancer‐specific death, and this association was independent of other potential prognostic factor. The prognostic influence of elevated LDH levels was consistent across all the LACC patient subgroups. Additionally, we found pretreatment LDH levels <252.0 *μ*/L was an independent predictor of CR after NACT.

Previous research has explored the biological mechanisms that are responsible for the association between elevated LDH levels and ominous prognosis in cancer patients. Possible explanations are as follows. First, tumor cells utilize glycolysis instead of mitochondrial oxidative phosphorylation to generate ATP that is required for the increased energy demand of the rapidly proliferating tumor cells 27. As a key enzyme in the process of glycolysis, LDH converts pyruvate and NADH to lactate and NAD+, determining the maintenance of glycolytic flow and consequently the production of ATP 28. Because LDH can be transcriptionally upregulated by hypoxia inducible factor 1*α* (HIF‐1*α*) and hypoxia in the tumor microenviroment is sufficient to stimulate the activation of HIF, there is a positive feedback loop between HIF and LDH under hypoxic conditions 29, 30. Therefore, elevated levels of LDH indicate an aggressive phenotype. Second, an increased serum LDH has been reported to reflect a heavy tumor burden 31, 32, 33. Owing to the heterogeneity of tumor cells, tumors with heavier load contain tumor cells with greater diversities 34. Thus, LDH‐positive patients are more susceptible to treatment resistance. Additionally, the vascular density is significantly higher in patients with elevated LDH levels which suggest an aggressive angiogenesis 35. As angiogenesis is essential for tumor proliferation and metastasis, patients with increased LDH levels are more likely to have a poor prognosis.

What is noteworthy is that cutoffs for LDH were heterogeneous in previous studies. In this study, we used 252.0 *μ*/L as the LDH cutoff. The impact of variations in LDH cutoffs has been evaluated in a published meta‐analysis 11. In the study, Zhang et al. pooled data from 68 studies and included 31,857 cancer patients. They concluded that high LDH is associated with an adverse prognosis in solid tumors and the variations in LDH cutoffs have no impact on its prognostic effect.

For patients with LACC, achieving optimal pathological response on surgical specimen is a strong predictor of good clinical outcome 36, 37. The study by Alessandro et al. 38 was the largest one to date that has assessed the benefit of NACT. Based on the long‐term follow‐up data (median follow‐up time: 12.7 years), the authors proposed response to NACT as a surrogate endpoint of survival for LACC patients. Given these findings and our own observations of the prognostic effect of CR for patients with LACC, we conducted an additional multivariate analysis and found that pretreatment LDH levels ≥252.0 *μ*/L was independently associated with decreased likelihood of CR after NACT (OR, 0.36; 95% CI, 0.23–0.57; *P *<* *0.0001). The result is in line with previous reports that showed LDH is a marker of response to NACT for breast cancer patients and oral cancer patients 23, 39. In vitro studies observed LDH is involved in resistance to chemotherapy, which may be an interpretation for the difference in CR rates by LDH levels 40, 41.

This study have several strengths including: (1) it was not only the first one to specifically explore the prognostic value of LDH in LACC but also the largest one to test the prognostic value of LDH in patients with gynecologic cancer; (2) all patients were newly diagnosed, so possible influence from disproportionate pretreatment that patients might receive can be ruled out; (3) all patients were from a single institution, so uniform treatment protocol can be ensured.

This study had several limitations. First, its observational design prevents us from discounting completely any residual factors of confusion that may influence the levels of LDH such as bone disease. Second, data about serial dynamic serum LDH levels are lacking. Finally, the findings of this study may be specific to Asian populations.

## Conclusion

In summary, our study suggests that baseline LDH ≥252.0 *μ*/L is an independent prognostic predictor for LACC patients treated with NACT. Furthermore, LACC patients with LDH levels ≥252.0 *μ*/L are less likely to achieve CR after NACT. Further study with adequate statistical power is needed to confirm and validate our findings. If validated, baseline LDH, an inexpensive and readily available laboratory parameter, could be utilized as a biomarker that can help physicians further categorize LACC patients with different prognosis and define the appropriate patient subgroup for NACT.

## Conflict of Interest

The authors declare that there are no conflicts of interest.

## Ethical Approval

The study complied with the Declaration of Helsinki and was approved by the Medical Ethics Committee of Sun Yat‐Sen Memorial Hospital, Sun Yat‐Sen University.
